# The Successful Use of Noninvasive Ventilation in Severe Asthma Exacerbation: A Case Report and Review of the Literature

**DOI:** 10.7759/cureus.107025

**Published:** 2026-04-14

**Authors:** Sakar B Gharti, Ernestine Faye S Tan, Marie Schmidt

**Affiliations:** 1 Internal Medicine, Interfaith Medical Center, New York, USA; 2 Pulmonary and Critical Care Medicine, Interfaith Medical Center, New York, USA

**Keywords:** asthma, bipap, critical care, hypercapnia, hypoxia, intubation, niv, non-invasive ventilation (niv), pulmonary, respiratory failure

## Abstract

Acute exacerbations of bronchial asthma (AEBA) complicated by hypercapnic respiratory failure (RF) are traditionally managed with invasive mechanical ventilation (IMV) when medical therapy fails. However, IMV is associated with significant complications, including barotrauma, hemodynamic instability, and increased mortality. The role of noninvasive ventilation (NIV) in AEBA remains controversial due to the limited and inconsistent nature of the available evidence.

We report the case of an 18-year-old female with a history of bronchial asthma who presented with severe AEBA and acute hypercapnic RF that was refractory to standard medical therapy, including bronchodilators, systemic corticosteroids, epinephrine, and magnesium sulfate. Arterial blood gas (ABG) analysis demonstrated respiratory acidosis, with a peak partial pressure of carbon dioxide (PaCO_2_) of 58 mmHg and a pH of 7.21. Although she was markedly tachypneic and in significant respiratory distress, she remained alert and hemodynamically stable, which allowed for a closely monitored trial of bilevel positive airway pressure (BiPAP) in the ICU. Rapid improvement in gas exchange was observed within three hours, with complete normalization by 10 hours, thereby avoiding the need for endotracheal intubation.

## Introduction

Bronchial asthma (BA) is a chronic, reversible obstructive airway disease characterized by episodic airway inflammation, bronchospasm, mucus hypersecretion, and airway edema [1,2]. In 2022, approximately 44.5 million individuals in the United States were living with asthma, representing a 48% increase in prevalence compared to prior estimates [3]. Annually, asthma exacerbations account for an estimated two million emergency department (ED) visits and approximately 500,000 hospitalizations, highlighting the substantial clinical and economic burden of the disease [2].

Acute exacerbations of bronchial asthma (AEBA) complicated by respiratory failure (RF) present substantial challenges in clinical management. Patients with AEBA commonly develop hypoxic respiratory failure; however, in cases of severe bronchospasm that do not respond to standard therapy, acute hypercapnic respiratory failure may ensue, which typically constitutes an indication for invasive mechanical ventilation (IMV). Intubation and mechanical ventilation are associated with significant complications, including barotrauma, hemodynamic instability, and increased mortality. For these reasons, there has been a growing trend toward the use of noninvasive ventilation (NIV) in AEBA, particularly in patients with mild to moderate hypercapnia. However, its application in this setting remains controversial, largely due to limited and inconsistent evidence.

NIV is widely used in the management of acute hypercapnic RF secondary to acute exacerbations of chronic obstructive pulmonary disease (AECOPD), hypoxic RF associated with congestive heart failure (CHF), and acute respiratory distress in immunocompromised patients [1,2]. While NIV is not routinely recommended for AEBA with hypercapnic RF, emerging literature suggests that it may be cautiously considered in selected patients [4,5]. As NIV is well established as the first-line therapy for hypercapnic RF in AECOPD, several studies propose that NIV may play a comparable role in managing hypercapnic respiratory failure during AEBA, with potential for safe application [4,5]. Studies have suggested that NIV can be used to support respiration, improve gas exchange, reduce the work of breathing, and decrease CO₂ retention in carefully selected patients experiencing acute asthma exacerbations [3].

In this report, we present a case demonstrating the successful use of NIV in a patient with AEBA complicated by hypercapnic respiratory failure. We describe the clinical factors that were associated with successful NIV in this patient. By comparing our findings with previously published evidence, including studies examining predictors of NIV success and failure, we aim to further discuss how patient and clinical characteristics are associated with favorable outcomes and add to the growing body of evidence supporting the careful and appropriate use of NIV in AEBA.

## Case presentation

An 18-year-old female with a diagnosis of BA presented to the ED with shortness of breath (SOB). She had a past medical history of iron deficiency anemia, pre-diabetes, and obesity, with a BMI of 32 kg/m². She had been diagnosed with BA in childhood and had allergies to peanuts and fish. Her common asthma triggers were dust mites. For her asthma exacerbations, she would often receive short courses of prednisone tablets and nebulization treatments, but she had never been admitted to the hospital. Her last asthma attack had been one month prior, and she had been discharged on fluticasone 110 mcg, two puffs twice daily, as her controller medication. She did not experience activity limitations or nighttime awakenings and used her rescue albuterol inhaler approximately once every one to two weeks as needed. She also had atopic dermatitis, for which she used topical mometasone cream.

On the day of admission, she experienced SOB that was not relieved by her short-acting albuterol inhaler, and an ambulance was called. On arrival to the ED, her oxygen saturation (SpO₂) was low at 89%. She received two doses of salbutamol-ipratropium (Duoneb) nebulization, dexamethasone 12 mg, two doses of intramuscular epinephrine, and one dose of magnesium sulfate. Despite medical treatment, she remained short of breath, tachycardic at 122 beats per minute, tachypneic at 38 breaths per minute, and was only able to speak in short sentences. Her blood pressure was 160/97 mmHg, and she was afebrile on arrival.

On examination, she had wheezing throughout both lung fields, decreased air entry, and was using accessory muscles to breathe. Her laboratory findings are presented in Table 1. Arterial blood gas (ABG) on admission showed a partial pressure of carbon dioxide (PCO₂) of 51.5 mmHg. PCO₂ peaked at 58 mmHg after two hours, as shown in Table 2. Her pH was also low at 7.25, and bicarbonate was low, indicating some degree of metabolic compensation. Her chest X-ray was normal, and her electrocardiogram showed sinus tachycardia. She remained in severe distress but was alert and oriented.

**Table 1:** Laboratory results on admission

Test	Normal value	Patient value
Hemoglobin	11.4 - 15.5 g/dL	11.0
Hematocrit	37 - 43.7%	35.0
Mean corpuscular volume	82 - 94.5 fL	74.5
Mean corpuscular hemoglobin	26.9 - 32.5 pg	23.3
Red cell distribution index	12.6 - 14.9%	27.4
White blood cells	4.5 - 10.2 x 10^3^/uL	10.9
Neutrophils	47.9 - 74.1%	79.7
Lymphocytes	17.8 - 41.1%	9.6
Basophils	0.3 - 1.1%	0.7
Eosinophils	0.1 - 4.7%	3.0
Monocytes	4.5 - 10.7%	7.0
Blood urea nitrogen	7 - 25 mg/dL	7.0
Creatinine	0.6 - 1.2 mg/dL	0.6
Estimated glomerular filtration rate	*≥*90.0 mL/min/1.73m^2^	133
Sodium	136 - 145 mEq/L	138
Potassium	3.5 - 5.1 mEq/L	3.9
Chloride	98 - 107 mEq/L	105.0
Bicarbonate	21 - 31 mEq/L	21.0
Calcium	8.6 - 10.3 mg/dL	9.7
Albumin	3.6 - 5.0 g/dL	4.8
Alanine aminotransferase	7 - 52 U/L	3.0
Aspartate aminotransferase	13 - 39 U/L	24.0
Magnesium	1.9 - 2.7 mg/dL	2.9
Phosphorus	2.5 - 5.0 mg/dL	4.2
Glucose	70 - 99 mg/d	134

**Table 2:** Arterial blood gas results on admission and after NIV NIV: non-invasive ventilation; FiO_2_: sraction of inspired oxygen; BiPAP: bilevel positive airway pressure; LPM: liters per minute; NC: nasal cannula; pCO_2_: partial pressure of carbon dioxide; pO_2_: partial pressure of oxygen; HCO_3_: bicarbonate, O_2_ sat: oxygen saturation

Variable	Normal values	0 hours	2 hours	5 hours	12 hours
NIV FiO_2_	-	N/A, room air FiO_2_ 21%	BiPAP FiO_2_ 50%	BiPAP FiO_2_ 40%	2 LPM per NC FiO_2_ 28%
pH	7.35 - 7.45	7.25	7.21	7.25	7.37
pCO_2_	35 - 45 mmHg	51.5	58	46.3	36
pO_2_	80 - 110 mmHg	90.1	305	149	93
HCO_3_	22 - 26 mmol/L	21	22	19.6	20.5
O_2_ sat	95 - 100%	95.3	>99%	98.5%	97.7

The patient was compliant with treatment and remained awake, alert, and oriented to three spheres. Due to her respiratory acidosis and willingness to comply with treatment, a decision was made to initiate a trial of NIV with bilevel positive airway pressure (BiPAP), with an inspiratory positive airway pressure (IPAP) of 14, an expiratory positive airway pressure (EPAP) of 5, and a fraction of inspired oxygen (FiO₂) of 50%. She was admitted to the ICU for close monitoring under the care of a critical care team due to the risk of deterioration that might require intubation. Medical management consisting of steroids and Duoneb was continued, and antibiotics (ceftriaxone and doxycycline) were initiated empirically while waiting for laboratory results.

Her blood gas improved over the next three hours and returned to normal within 10 hours of starting NIV. FiO₂ progressively decreased to 28%, as shown in Table 2. Due to the lack of evidence of active infection, antibiotics were subsequently discontinued. She was downgraded to the regular floor on hospital day two. Intravenous methylprednisolone was transitioned to oral prednisone, and the patient was discharged on hospital day three with follow-up arranged at the outpatient pulmonary clinic.

## Discussion

Management of AEBA involves supplemental oxygen and pharmacologic therapy, including inhaled bronchodilators, systemic glucocorticoids, magnesium sulfate, and anticholinergic agents [2,4-5]. While many patients respond to these interventions, a subset fails to demonstrate adequate clinical improvement and may require IMV, especially those with hypercapnic RF [3,4]. Endotracheal intubation and IMV are associated with significant complications, including cardiovascular collapse related to sedative-induced vasodilation, increased intrathoracic pressure leading to reduced cardiac output, and procedural risks such as arrhythmias, hypotension, and cardiac arrest [6-10]. Additional adverse outcomes include barotrauma, increased risk of infection, aspiration, and prolonged ICU and hospital length of stay [6-10]. Pallin and Naughton demonstrated that patients requiring IMV during an acute asthma exacerbation experienced a threefold increase in mortality compared with those managed without IMV [8]. Therefore, strategies aimed at avoiding IMV are of paramount importance in the management of severe asthma exacerbations.

Although consensus guidelines have not formally endorsed NIV for acute asthma exacerbations, multiple studies have reported favorable outcomes. A 2024 systematic review incorporating five randomized controlled trials and eight observational studies found that NIV use in acute asthma was associated with reduced rates of intubation and hospital admission, as well as faster improvement in accessory muscle use, compared with standard medical therapy alone [1]. These findings support consideration of a monitored trial of NIV in patients presenting with severe asthma exacerbations. Similar outcomes have been reported in larger observational and prospective studies. A retrospective cohort study of 53,654 patients across 682 U.S. hospitals between 2010 and 2017 found that NIV use was associated with significantly lower odds of progression to IMV and reduced in-hospital mortality [2]. In a prospective study by Meduri et al., NIV was associated with reductions in respiratory muscle use, dyspnea, and respiratory rate, without complications related to secretion clearance or oral intake [11].

Talbot et al. reported a decline in the use of IMV, from 1.4% in 2000 to 0.73% in 2008, accompanied by a nearly fivefold increase in the use of NIV [12]. These findings were corroborated by a subsequent study conducted by Stefan et al. across 97 hospitals, involving 13,930 patients hospitalized with acute asthma exacerbations. In this cohort, more than 40% of patients received non-invasive ventilation, reflecting a substantial increase in NIV utilization [13]. The physiological rationale supporting NIV use in acute asthma includes reduction of the work of breathing, promotion of alveolar recruitment, improvement in dynamic lung compliance, offsetting intrinsic positive end-expiratory pressure (PEEP), and enhancement of gas exchange during acute respiratory failure [11-14].

Stefan et al. proposed three factors contributing to the increasing use of NIV in acute asthma [13]. First, clinicians may extrapolate from the pathophysiologic similarities between acute asthma exacerbations and COPD exacerbations, particularly with respect to bronchospasm and dynamic hyperinflation [13]. Second, increased familiarity and comfort among physicians and respiratory therapists with NIV have likely facilitated its application beyond traditionally evidence-supported indications [13]. Third, the expanding use of NIV outside the intensive care unit may further encourage its adoption in patients with less severe forms of respiratory failure [13].

Predictors of NIV failure in asthma remain incompletely defined. Ganesh et al. evaluated 98 patient encounters with asthma exacerbations and found no significant differences in age, sex, race, smoking status, BMI, baseline blood gas values, asthma severity, or prior ICU admission between patients with successful versus failed NIV [15]. Among the 19 patients who failed NIV, higher initial oxygen requirements were associated with an increased likelihood of subsequent intubation [15]. In a large multicenter study by Stefan et al., 4.7% of patients initially managed with NIV required subsequent intubation [13]. Predictors of subsequent intubation after an NIV trial included prior hospitalization for asthma within 12 months, diabetes mellitus, and concomitant pneumonia [13]. Other predictors of potential NIV failure are listed by Farmer and associates as: inability to protect the airway, hypotension, a Glasgow Coma Scale score of less than 11, severe agitation, profuse secretions, acute respiratory distress syndrome (ARDS), pH less than 7.25, tachypnea greater than 30 breaths per minute, age above 40 years, asynchronous breathing, shock, edentulism, and an Acute Physiology and Chronic Health Evaluation (APACHE) II score of 29 or more [16].

Delayed recognition of NIV failure and postponement of intubation are associated with poorer outcomes [16]. Appropriate patient selection requires a multidisciplinary team that can recognize signs of NIV failure and contraindications to an NIV trial at the outset, and an experienced clinical team is crucial for success. With this in mind, our patient was closely monitored in the ICU setting, as she had one predictor of NIV failure: tachypnea of 38 breaths per minute. Continuous oxyhemoglobin saturation monitoring and objective evaluation of airflow obstruction are crucial in such cases [17].

To summarize, review of the aforementioned literature suggests that NIV may be considered a reasonable therapeutic option in select patients with AEBA and hypercapnic respiratory failure, provided the following conditions are met: (1) hypercapnic respiratory failure is mild to moderate; (2) the patient is alert, cooperative, and hemodynamically stable; (3) there is no evidence of impending respiratory arrest; (4) rapid access to endotracheal intubation and ICU support is available; and (5) close monitoring is ensured by experienced staff.

Our case further illustrates a potential benefit of NIV at a point when progression to IMV appeared imminent, resulting in rapid clinical improvement, prevention of further respiratory deterioration, and a shortened hospital course. Using literature data as stated above, a decision was made to initiate an NIV trial, which proved to be successful. The patient demonstrated marked improvement, both clinically and objectively, as reflected by improved ABG values within three hours and normalization within 10 hours after starting NIV. She was discharged the following day, thereby avoiding the risks associated with IMV and prolonged hospitalization. However, a key limitation is that a case report cannot be generalized to a broader population; therefore, independent NIV trials should be interpreted with caution, considering both existing literature and these findings.

## Conclusions

NIV may be considered a reasonable trial in carefully selected individuals experiencing acute asthma exacerbations, especially those with mild to moderate hypercapnia, intact mental status, and hemodynamic stability, provided that close monitoring and immediate availability of intubation are ensured. Prompt identification of NIV failure, such as worsening acidosis, including rising PaCO₂, deterioration in mental status, or respiratory fatigue, is essential because delays in intubation are linked to worse outcomes. NIV should not substitute invasive mechanical ventilation when clinically indicated. Additional large-scale randomized controlled trials are required to better delineate the role of NIV in acute asthma care, particularly regarding its effects on intubation rates and mortality.
